# 3D Porous VO_x_/N-Doped Carbon Nanosheet Hybrids Derived from Cross-Linked Dicyandiamide–Chitosan Hydrogels for Superior Supercapacitor Electrode Materials

**DOI:** 10.3390/polym15173565

**Published:** 2023-08-28

**Authors:** Jinghua Liu, Xiong He, Jiayang Cai, Jie Zhou, Baosheng Liu, Shaohui Zhang, Zijun Sun, Pingping Su, Dezhi Qu, Yudong Li

**Affiliations:** 1 Liuzhou Key Laboratory of New Energy Vehicle Power Lithium Battery, Guangxi Engineering Research Center for Characteristic Metallic Powder Materials, School of Electronic Engineering, Guangxi University of Science and Technology, Liuzhou 545000, China; liujinghua@gxust.edu.cn (J.L.); 17586600924@163.com (J.Z.); liubaosheng@gxust.edu.cn (B.L.); zhangshaohui@gxust.edu.cn (S.Z.); sunzijun@gxust.edu.cn (Z.S.); 2 Guangxi Key Laboratory of Green Processing of Sugar Resources, College of Biological and Chemical Engineering, Guangxi University of Science and Technology, Liuzhou 545006, China; cjy193677464@163.com (J.C.); m19807720904@163.com (P.S.); 3 Key Laboratory of Bio-Based Material Science & Technology, Northeast Forestry University, Harbin 150090, China; lydlnn0000@163.com

**Keywords:** 3D structure, porous carbon, supercapacitor, heteroatom-doping

## Abstract

Three-dimensional porous carbon materials with moderate heteroatom-doping have been extensively investigated as promising electrode materials for energy storage. In this study, we fabricated a 3D cross-linked chitosan-dicyandiamide-VOSO_4_ hydrogel using a polymerization process. After pyrolysis at high temperature, 3D porous VO_x_/N-doped carbon nanosheet hybrids (3D VNCN) were obtained. The unique 3D porous skeleton, abundant doping elements, and presence of VO_x_ 3D VNCN pyrolyzed at 800 °C (3D VNCN-800) ensured excellent electrochemical performance. The 3D VNCN-800 electrode exhibits a maximum specific capacitance of 408.1 F·g^−1^ at 1 A·g^−1^ current density and an admirable cycling stability with 96.8% capacitance retention after 5000 cycles. Moreover, an assembled symmetrical supercapacitor based on the 3D VNCN-800 electrode delivers a maximum energy density of 15.6 Wh·Kg^−1^ at a power density of 600 W·Kg^−1^. Our study demonstrates a potential guideline for the fabrication of porous carbon materials with 3D structure and abundant heteroatom-doping.

## 1. Introduction

Chitosan (CS), as an eco-friendly biopolymer, has drawn considerable attention in the fabrication of various materials [1,2,3]. The presence of -NH_2_ and -OH groups in chitosan molecules makes them easily polymerized. With the assistance of glutaraldehyde, a 3D cross-linked gel can form through the interaction between amino groups in chitosan molecules and aldehyde functional groups in glutaraldehyde molecules [4]. The unique 3D structure was well maintained after carbonizing at high temperatures and leaves a 3D carbon skeleton. Moreover, the original N species in chitosan can introduce N-doping elements into the carbon skeleton. The gas released during the pyrolysis process ensures the carbon material’s porous structure. Without post-treatment processing, this efficient preparation method has recently been adopted to fabricate 3D carbon materials.

Carbon materials, as considerable supercapacitor electrode materials, have been extensively investigated to improve their specific capacitance [5,6,7,8]. Structure engineering is necessary for optimizing the carbon materials used in supercapacitors [9,10]. Three-dimensional porous structures have acquired wide attention due to high specific surface areas and large numbers of pores, which provide fast ion/electron transport [11,12,13]. These 3D porous carbon materials are also of great importance in designing advanced architecture for micro-supercapacitors [14]. Recently, the “bombing effect” method has been adopted to synthesize 3D porous carbon materials derived from popcorn, rice husk, etc. [15,16,17]. In addition, this polymerization method was also used to build 3D structures upon polymerizing various molecules. After pyrolysis at high temperatures, the 3D structure is well maintained, and large numbers of pores are formed simultaneously. For instance, Tong’s group fabricated 3D porous carbon/graphene hybrids through pyrolyzing graphene oxides-CS hydrogels, which exhibited an enhanced specific capacitance of 320 F·g^−1^ at 1 A·g^−1^ [4]. Zhou’s group prepared N and O enriched hierarchical porous carbon derived from CS-based hydrogel beads by microwave heating, which shows remarkable rate capability [18].

Heteroatom-doping is another effective method to improve specific capacitance of carbon materials [19,20]. The faradaic reaction at doping sites contributes additional pseudocapacitance to supplement electric double layer capacitance. To date, N, S, P, and B doping have been proved as effective methods to improve specific capacitance of carbon materials. Among them, the N-doping method has been verified as an effective approach to improve capacitance via surface faradaic reactions without sacrificing rate performance and cycle stability [12,21,22,23]. In addition to introducing doping elements, integrating suitable pseudo-capacitive materials with carbon materials is also widely used to improve electrochemical performance of carbon materials. Vanadium-based nanocomposites are promising energy storage materials. For instance, vanadium oxides have the merits of versatile structure, high capacity, easy synthesis, and adequate safety, which is widely used in various types of energy storage devices, such as Li-ion batteries, Na-ion batteries, K-ion batteries, and supercapacitors [24,25]. A hybrid of VN nanoparticle-assembled hollow microspheres encapsulated in N-doped nanofibers has been fabricated by Yang’s group, which exhibits a superb rate property and prolonged cyclability as the anode in K-ion battery [26]. A novel polyoxovanadate-based metal-organic framework microsphere with good supercapacitor performance was synthesized by Guo’s group [27]. A comparative list of performance metrics for 3D porous carbon materials, 3D N-doped porous carbon materials, and 3D porous carbon materials combined with pseudocapacitive materials is shown in Table S1. It can be seen that introducing doping elements and pseudocapacitive materials are efficient approaches to improving the capacitive performance of carbon materials.

Herein, we design a new route to fabricate 3D porous VO_x_/N-doped carbon nanosheet hybrids (3D VNCN) through the pyrolysis of cross-linked chitosan-dicyandiamide-VOSO_4_ (CS-DCDA-VOSO_4_) hydrogel. The aldehyde functional groups in glutaraldehyde can polymerize with -NH_2_ and -OH functional groups from CS and DCDA, leading to a cross-linked network structure. Meanwhile, VOSO_4_ uniformly disperses within the hydrogel. After pyrolysis at high temperature, 3D VNCN hybrids were obtained. The unique 3D skeleton, abundant doping elements, porous structure, and presence of VO_x_ ensure the excellent electrochemical performance of 3D VNCN. The 3D VNCN electrode exhibits a maximum specific capacitance of 408.1 F·g^−1^ at 1 A·g^−1^ current density. The maintained capacitance retention was about 96.8% after 5000 cycles. Moreover, a 3D VNCN-based symmetrical supercapacitor shows a maximum energy density of 15.6 Wh·Kg^−1^ at a power density of 600 W·Kg^−1^. It is worth noting that our study provides a promising method for the fabrication of 3D porous carbon materials used in the energy storage field.

## 2. Materials and Methods

### 2.1. Preparation of 3D VNCN

In a typical procedure, 0.2 g of CS, 0.3 g of DCDA, and 0.05 g of VOSO_4_ were dissolved in 20 mL deionized water. A volume of 200 μL of acetic acid was added dropwise into the above solution and stirred for 15 min. Then, 400 μL glutaraldehyde was added into the above homogeneous suspension for polymerization. After standing for 2 h, the resulting jelly-like hydrogel containing CS, DCDA, and VOSO_4_ (CS-DCDA-VOSO_4_ hydrogel) was obtained. Followed by freeze–drying for 24 h, the hydrogel transformed into a 3D interconnected porous structure, and then the dried CS-DCDA-VOSO_4_ gel was transferred to a tube furnace for further carbonization. The carbonization process was adopted using a two-step pyrolysis method under N_2_ atmosphere at a heating rate of 5 °C·min^−1^. At first, the temperature was increased to 600 °C and held for 2 h, and then increased to a higher temperature (700 °C, 800 °C, and 900 °C) for 3 h. The products were marked as 3D VNCN-700, 3D VNCN-800, and 3D VNCN-900, respectively. For comparison, 3D N-doped carbon nanosheets derived from CS-DCDA hydrogel at 800 °C were denoted as 3D NCN. CS, DCDA, and VOSO_4_ with the same ratio were ground into a light blue powder (CS-DCDA-VOSO_4_ powder). After pyrolyzing at 800 °C, the obtained VO_x_/N-doped carbon material was marked as VNC.

### 2.2. Materials Characterization

X-ray diffraction (XRD, Ultima IV, RIGAKU, Tokyo, Japan) was conducted with Cu-Ka radiation (λ = 0.1504 nm) to investigate the phase of the as-prepared samples. Morphologies and structures of the samples were characterized using a scanning electron microscope (SEM, Gemini 300, Zeiss, Jena, Germany), transmission electron microscope (TEM, Tecnai F20, FEI, Hillsboro, OR, USA), and X-ray photoelectron spectroscope (XPS, Scientific K-Alpha+, Thermo fisher, Waltham, MA, USA). N_2_ adsorption and desorption isotherms were carried out on a Micrometrics ASAP 2020 V3.04 H system with Brunauer–Emmett–Teller measurements (BET). The functional groups on the surface of the samples were obtained using Fourier transform infrared (FT-IR) spectroscopy with a Nicolet 50 spectrometer. Raman measurements were investigated via an inVia confocal micro-Raman spectroscope (RTS2). Thermogravimetric analysis (TGA) was performed on Discovery TGA5500 (TA, New Castle, DE, USA) at a heating rate of 5 °C·min^−1^ under flowing N_2_.

### 2.3. Electrochemical Measurement

The electrochemical performance was characterized using a CHI760E electrochemical workstation. In a three-electrode system, Pt foil and a Hg/HgO electrode were used as counter and reference electrodes, respectively. The working electrode was prepared by coating a slurry on Ni foam. The slurry was prepared by mixing the active material with acetylene black and polytetrafluoroethylene in ethanol solvent at a mass ratio of 8:1:1. After being pressed under 10 MPa for 30 s and dried at 100 °C for 12 h, the working electrode was obtained for further electrochemical characterization. The mass loading of active material in the working electrode was about 2 mg. A 6 M KOH aqueous solution was used as the electrolyte. Cyclic voltammetry (CV) and galvanostatic charge–discharge (GCD) curves were obtained in the potential range of −0.95–0.25 V. Electrochemical impedance spectroscopy (EIS) measurements were tested under open circuit voltage at an amplitude of 5 mV with a frequency range between 0.01 and 100 kHz. The specific capacitance of the active material (*Cs*, F·g^−1^) was calculated from the GCD curves according to the equation *Cs* = (*I* × ∆*t*)/(*m* × ∆*V*), where *I* (A) is the discharge current, ∆*t* (s) is the discharge time, *m* (g) is the mass of the active material, and ∆*V* (V) is the voltage change during the discharge process. Furthermore, a symmetric supercapacitor was assembled with two identical electrodes, and the device was characterized using a 6 M KOH aqueous solution as the electrolyte. The energy and power density were characterized using the following equation: *E*_cell_ = *C*_cell_ × ∆*V^2^*/7.2 and *P*_cell_ = *E*_cell_ × 3600/∆*t.*

## 3. Results

### 3.1. Characterization of Prepared Materials

**Figure 1:**
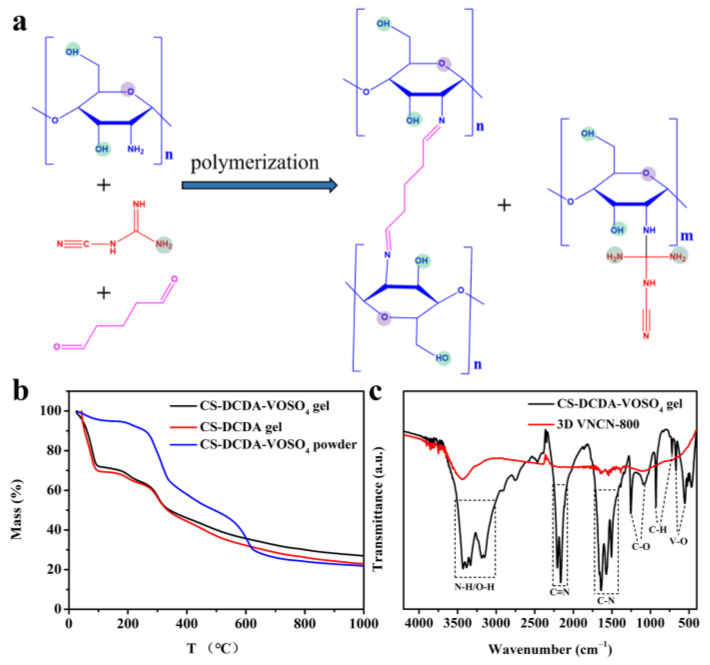
(**a**) Illustration of the polymerization process, (**b**) TGA curves of CS-DCDAVOSO_4_ gel, CS-DCD gel, and CS-DCDA-VOSO_4_ powder, (**c**) FT-TR spectra of CS-DCDA-VOSO_4_ gel and 3D VNCN-800.

The fabrication of 3D VNCN adopted a two-step method. At first, CS and DCDA were polymerized with the assistance of glutaraldehyde and formed a transparent hydrogel, as illustrated in Figure 1a. During this procedure, a 3D cross-linked structure was fabricated through a synergistic process that included the interaction between the amino groups in CS and aldehyde functional groups in glutaraldehyde molecules, and amination reaction between CS and DCDA, as well as hydrogen bonding among CS [28,29]. Meanwhile, VOSO_4_ was uniformly dispersed into the hydrogel and formed CD-DCDA-VOSO_4_ hydrogel. After freezing–drying for 24, the dried gel was pyrolyzed at different temperatures under an N_2_ atmosphere, and finally, the 3D VNCN composites were obtained.

TGA curves of CS-DCDA-VOSO_4_ gel, CS-DCDA gel, and CS-DCDA-VOSO_4_ powder were performed to simulate the pyrolysis process at N_2_ flow from room temperature to 1000 °C as presented in Figure 1b. It can be seen that TGA curves of CS-DCDA-VOSO_4_ gel and CS-DCDA gel undergo almost similar mass loss process, demonstrating that a small amount of VOSO_4_ has negligible influence on the pyrolysis process. Before 80 °C, their TGA curves exhibit severe mass loss due to the loss of large amounts of water in CS-DCDA-VOSO_4_ gel and CS-DCDA gel. In the next stage, the mass loss can be attributed to the loss of crystal water and small molecule species. Upon increasing the temperature, the polymerized CS-DCDA gel decomposes to an N-doped carbon material. While VOSO_4_ gradually transforms to VO_x_ and VO_x_ are decorated on carbon skeleton, leading to VO_x_/N-doped carbon hybrids. Compared with CS-DCDA-VOSO_4_ gel and CS-DCDA gel, CS-DCDA-VOSO_4_ powder contains a small quantity of water content. The lack of a polymerization process between CS and DCDA makes the TGA curves slightly different. The mass loss in the first stage (<220 °C) also results from the loss of crystal water and small molecule species. During the next stage, CS decomposes to a carbon material, and DCDA successively condenses to melamine, tris-s-triazine, and C_3_N_4_ from 220 °C to 600 °C. VOSO_4_ transforms to VO_x_ at this stage. Then, at higher temperatures (>600 °C), C_3_N_4_ further decomposes and leaves N species introduced into carbon materials derived from chitosan. VO_x_ nanoparticles are also decorated on carbon skeleton, producing a VNC composite.

The FT-IR spectra (Figure 1c) were used to characterize the functional groups of CS-DCDA-VOSO_4_ gel and 3D VNCN-800. It can be observed that the FT-IR spectrum of CS-DCDA-VOSO_4_ gel exhibits obvious characteristic peaks ranging from 4200 cm^−1^ to 800 cm^−1^. Among them, the characteristic peaks from 3600 to 3000 cm^−1^ are attributed to the stretching vibration absorption peak of the N–H and O–H groups [30]. The appearance of strong peaks from 2300 to 1900 cm^−1^ is ascribed to C≡N stretching modes. The peaks in the wave number range of 1800–1300 cm^−1^ are caused by C–N heterocyclic stretching vibrations [31]. The peaks at 1254 and 1078 cm^−1^ are indexed to C–O stretching modes [32]. The peaks at 560 and 665 cm^−1^ refer to V–O symmetric and stretching vibrations [33,34]. After pyrolysis at 800 °C, most functional groups are eliminated; thus, the FT-IR spectrum of 3D VNCN-800 only retains a distinct O–H stretching vibration absorption peak around 3400 cm^−1^. No obvious peak of V–O bonds can be observed due to the low content of VO_x_.

**Figure 2:**
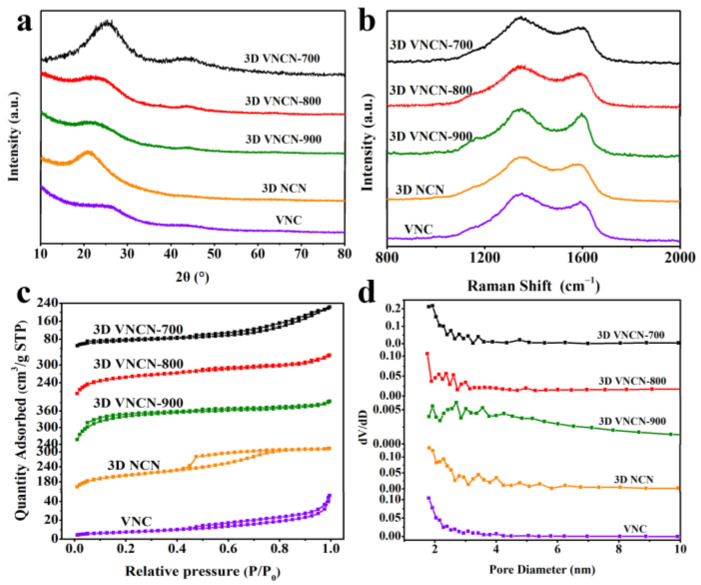
(**a**) XRD patterns of 3D VNCN, 3D NCN, and VNC, (**b**) Raman spectra of 3D VNCN, 3D NCN, and VNC, (**c**) N_2_ adsorption/desorption isotherms of 3D VNCN, 3D NCN, and VNC, (**d**) PSD curves of 3D VNCN, 3D NCN, and VNC.

Figure 2a shows the XRD patterns of 3D NCN, VNC, and 3D VNCN at different temperatures. Broad characteristic peaks centered around 24° and 44° correspond to the (002) and (100) planes of the graphitic plane, demonstrating the successful preparation of carbon materials. The (002) plane of 3D VNCN at different temperatures shows slight variation from 25.3° to 22.1°. Upon increasing the temperature, the 2*θ* degree of 3D VNCN gradually decreases, and the intensity of the (002) peak becomes weaker. This phenomenon can be ascribed to the increased temperature, which reduces stacking between carbon sheets, presenting enlarged interlayer spacing and loose structure of carbon materials. The (002) peak of 3D NCN and VNC centers at 21.2° and 26.6°, exhibiting distinct change compared with 3D VNCN. Without adding VOSO_4_, 3D NCN becomes less dense compared with 3D VNCN; thus, the (002) plane shifts to a low angle. While VNC fabricated from CS-DCDA-VOSO_4_ powder shows a bulk structure. The highly stacked structure results in the higher position of the (002) plane. Both VNC and 3D VNCN show no obvious VO_x_ diffraction peaks, which may be ascribed to the small quantity of VO_x_ in VNC and 3D VNCN. Raman characterizations were conducted to evaluate the graphitic degree of 3D VNCN, 3D NCN, and VNC in Figure 2b. Typical D-bands at around 1350 cm^−1^ are associated with disordered carbon structure. At the same time, G-bands at around 1600 cm^−1^ correspond to *sp^2^*-hybridized carbon [35,36]. The *I*_D_/*I*_G_ values of 3D VNCN-700, 3D VNCN-800, 3D VNCN-900, 3D NCN, and VNC are 1.29, 1.23, 1.08, 1.18, and 1.46, respectively. This result demonstrates that these carbon materials are disordered structures with partial graphitization. The elevated temperature of 3D VNCN leads to a higher degree of graphitization. The 3D VNCN-800 product, with moderate defects and graphitic carbon, could provide abundant ion storage sites and facilitate ion/electron transportation. As for 3D NCN, without doping element and VO_x_, it exhibits an ordered structure compared with 3D VNCN-800. While VNC shows the highest *I*_D_/*I*_G_ ratio, suggesting a highly disordered structure owing to the lack of a 3D skeleton resulting from CS-DCDA hydrogel.

Figure 2c shows N_2_ adsorption and desorption isotherms of 3D VNCN, 3D NCN, and VNC. A combination of Type-I and Type-IV with a slight hysteresis loop at high relative pressure (0.5–1.0) can be observed, indicating the co-existence of micropores and mesopores [37]. The specific surface areas (SSAs) of 3D VNCN-700, 3D VNCN-800, 3D VNCN-900, 3D NCN, and VNC were 125.5 m^2^·g^−1^, 288.0 m^2^·g^−1^, 358.2 m^2^·g^−1^, 285.5 m^2^·g^−1^, and 17.5 m^2^·g^−1^, respectively. The 3D VNCN-800 and 3D NCN products exhibit similar SSAs, while VNC displays a particularly small SSA due to the stacked structure. The SSA of 3D VNCN gradually increases with increasing temperature due to exfoliated carbon sheets and decreased stacking structure at high temperatures. Pore size distribution (PSD) curves in Figure 2d show the pore structure of 3D VNCN, 3D NCN, and VNC. The wide pore distribution range from 2 nm to 5 nm demonstrates hierarchical porous structure in 3D NCN and 3D VNCN, while VNC displays fewer pores. The presence of hierarchical pores in 3D VNCN helps to reserve electrolyte ions and shorten the ion/electron transport distance, which is beneficial for improving electrochemical performance [38].

**Figure 3:**
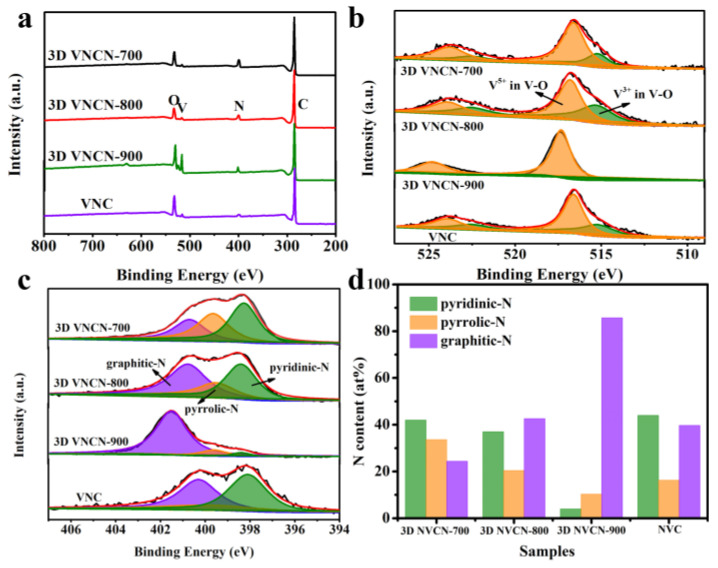
(**a**) XPS spectra of 3D VNCN and VNC, (**b**) V2p spectra of 3D VNCN and VNC, (**c**) N1s spectra of 3D VNCN and VNC, (**d**) the ratios of different nitrogen species determined from the N 1s XPS spectra.

The chemical compositions and surface element contents of 3D VNCN and VNC were investigated by XPS, and Figure 3a displays the corresponding XPS survey spectra. The strong signals reveal the co-existence of C, N, V, and O elements. The corresponding C, N, V, and O element contents are displayed in Table 1. The N content in 3D VNCN ranges from 9.05 to 4.61 %, which is higher than that of VNC. The phenomenon suggests that the 3D interconnected structure retains more N species after pyrolysis at high temperatures, which would be beneficial for improving the specific capacitance. Figure 3b displays the high-resolution V 2p peaks of 3D VNCN and VNC. Two sets of double peaks associated with V^3+^ in the V–O bond (515.2 eV and 522.3 eV) and V^5+^ in the V–O bond (516.6 eV and 523.8 eV) demonstrate the existence of the V element as vanadium oxide [39]. The co-existence of V^3+^ and V^5+^ in 3D VNCN could provide abundant faradic reaction sites, trigger fast redox reactions, and enhance specific capacitance. However, at 900 °C, the V species in 3D VNCN mainly comes from V^5+^. As for N1s, the high-resolution spectra (Figure 3c) exhibit three characteristic peaks associated with pyridinic-N (398.3 eV), pyrrolic-N (399.6 eV), and graphitic-N (400.6 eV), respectively [40]. The contents of different N species are illustrated in Figure 3d. It can be seen temperature greatly influences the content of pyridinic-N, pyrrolic-N, and graphitic-N. Upon increasing the temperature, the content of pyridinic-N and pyrrolic-N in 3D VNCN gradually decreases, while graphitic-N content increases. In addition, the content of N species in VNC is close to that of 3D VNCN-800 owing to the same pyrolysis temperature.

**Figure 4:**
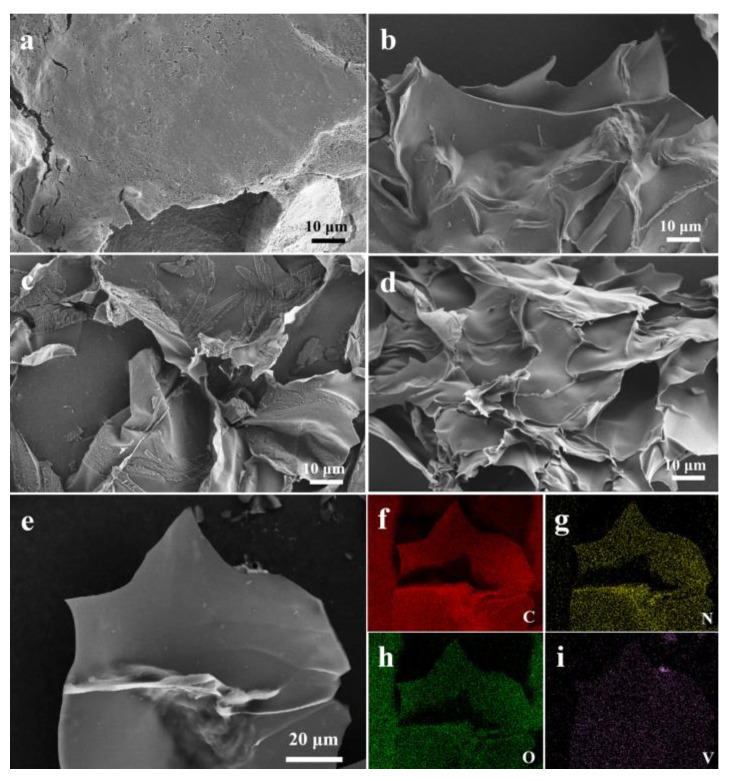
SEM images of (**a**) VNC, (**b**) 3D NCN, (**c**) CS-DCDA-VOSO_4_ gel, (**d**,**e**) 3D VNCN-800, (**f**–**i**) elemental mapping of C, N, O, and V.

Figure 4 shows the SEM images of VNC, 3D NCN, CS-DCDA-VOSO_4_ gel, and 3D VNCN-800. It can be seen the VNC composite (Figure 4a) shows a bulk structure with highly stacked sheets. While 3D NCN (Figure 4b), CS-DCDA-VOSO_4_ gel (Figure 4c), and 3D VNCN-800 (Figure 4d) display obvious inter-connected 3D porous structures formed from cross-linked carbon nanosheets, demonstrating that 3D inter-linked structure can be achieved through a polymerization process. The 3D cross-linked carbon skeleton facilitates conductivity and provides a fast ion/electron transport path, which could improve the electrochemical performance. In addition, the element mapping of C, N, O, and V elements in Figure 4f–i further demonstrates the successful formation of 3D VNCN.

**Figure 5:**
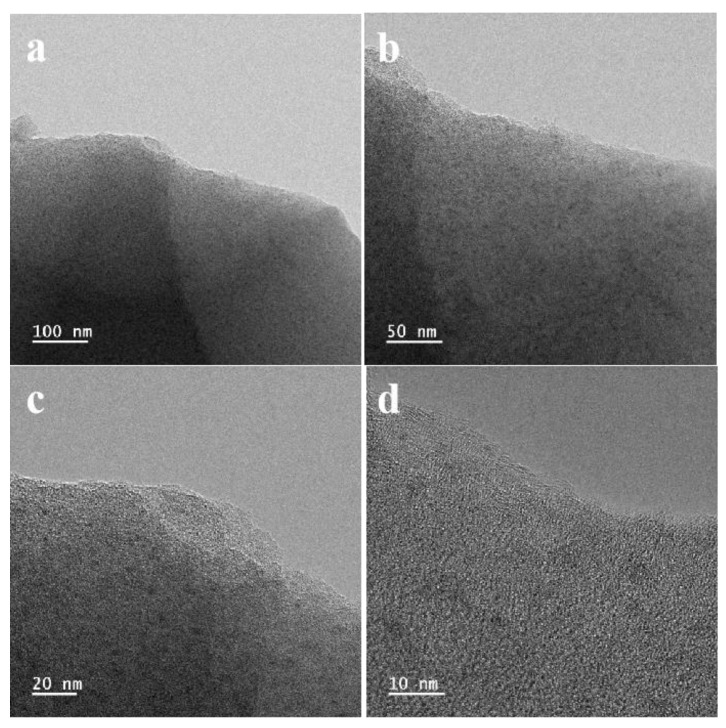
(**a**–**d**) TEM images of 3D VNCN-800.

TEM was carried out to investigate more detailed information on 3D VNCN-800. As shown in Figure 5, similar to the analysis from SEM, a layered structure rich in worm-like nanopores on the surface can be observed, demonstrating the porous structure. Such a porous structure can effectively shorten the ion-transport pathways and prevent restacking between carbon nanosheets. Moreover, it can also be observed that plenty of nanoparticles are dispersed on carbon nanosheets, which can be attributed to vanadium oxide. However, no obvious lattice fringe can be found, indicating the vanadium oxides are amorphous. This result agrees well with the XRD and XPS results.

### 3.2. Electrochemical Performance

**Figure 6:**
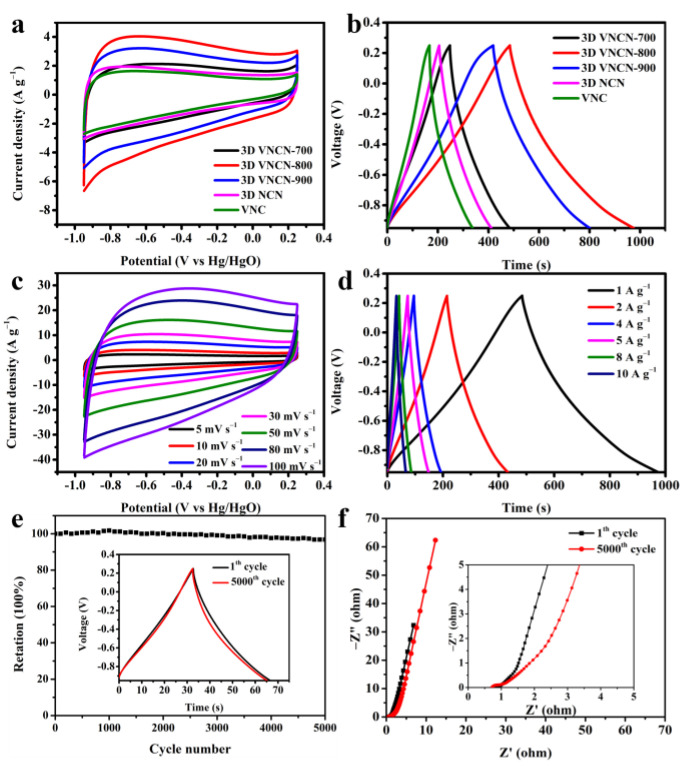
(**a**) CV curves of 3D VNCN, 3D NCN, and VNC at 10 mV·s^−1^. (**b**) GCD curves of 3D VNCN, 3D NCN, and VNC at 0.5 A·g^−1^. (**c**,**d**) CV and GCD curves of 3D VNCN-800 at different scan rates and different current densities. (**e**,**f**) Cycling performance and Nyquist plots of 3D VNCN-800.

The combination of 3D hierarchically porous structure with uniformly dispersed N-doping and VO_x_ nanoparticles makes 3D VNCN composites highly satisfactory as supercapacitor electrode materials. To evaluate the electrochemical performance of 3D VNCN, 3D NCN, and VNC electrodes, CV and GCD measurements were employed in a three-electrode system with 6.0 M aqueous KOH as the electrolyte. As shown in Figure 6a, all the CV curves exhibit nearly rectangular shapes, indicating good double-layer capacitive behavior. The quasi-triangular shapes of the GCD curves (Figure 6b) demonstrate the reversible Faradaic reactions and good conductivity. The longest discharge time indicates the highest specific capacitance of 3D VNCN-800. Calculated from the GCD curves in Figure 6b, the capacitance performances of 3D VNCN-700, 3D VNCN-800, 3D VNCN-900, 3D NCN, and VNC are summarized in Table 2. The highest specific capacitance of 3D VNCN-800 can be ascribed to the following reasons. (1) The inter-connected 3D porous structure facilitates conductivity and provides a fast ion/electron transport path. (2) The abundant N dopants provide large numbers of active sites to generate redox reactions and enhance specific capacitance. (3) The variable valence states of the V element in 3D NVCN-800 could trigger fast redox reactions and supplement additional pseudocapacitance.

Figure 6c shows the CV curves of the 3D VNCN-800 electrode at different scan rates. It can be seen the CV curve is well maintained even under 100 mV·s^−1^, indicating good rate capability. Figure 6d presents the GCD curves of the 3D VNCN-800 electrode at different current densities from 1 A·g^−1^ to 10 A·g^−1^. The calculated specific capacitances of the 3D VNCN-800 electrode are 408.1 F·g^−1^ (1 A·g^−1^), 365.0 F·g^−1^ (2 A·g^−1^), 324.0 F·g^−1^ (4 A·g^−1^), 313.3 F·g^−1^ (5 A·g^−1^), 290.7 F·g^−1^ (8 A·g^−1^), and 282.5 F·g^−1^ (10 A·g^−1^), respectively. The cycling stability of the 3D VNCN-800 electrode was evaluated using successive GCD measurements between −0.95 V and 0.25 V at 10 A·g^−1^. As shown in Figure 6e, the 3D VNCN-800 electrode exhibits admirable cycling stability with 96.8% capacitance retention after 5000 cycles. The recently reported 3D porous carbon-based electrode materials and their electrochemical performance investigated in three-electrode systems are summarized in Table S2. It is clear that our sample shows relatively high capacitive performances among these recently reported 3D porous carbon-based electrode materials.

EIS analyses were employed to evaluate the electron/ion transport process of the 3D VNCN-800 electrode before and after the cycling test. The Nyquist plot of the 3D VNCN-800 electrode (Figure 6f) shows a distorted semicircle in the high-frequency region and nearly vertical straight lines in the low-frequency zone, indicating low diffusion resistance and good EDLC performance [41,42]. The intercept from the x-axis represents the equivalent series resistance (*R*_s_), which is 0.68 Ω for the 3D VNCN-800 electrode. Moreover, the small semicircle from the Nyquist plot corresponding to charge transfer resistance indicates a fast adsorption/desorption rate for the 3D VNCN-800 electrode. After 5000 cycling tests, the Nyquist plot of the 3D VNCN-800 electrode shows negligible change, demonstrating well-maintained electrochemical performance.

**Figure 7:**
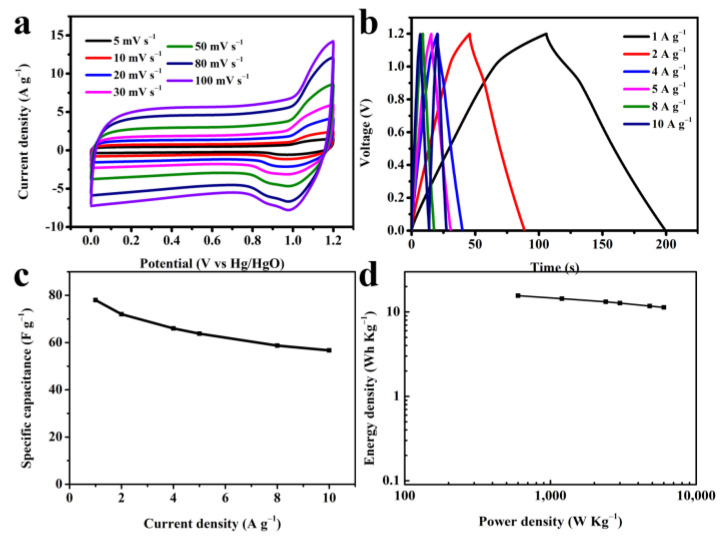
(**a**) CV curves of 3D VNCN-800-SC at different scan rates. (**b**) GCD curves of 3D VNCN-800-SC at different current densities. (**c**) Relationship between the specific capacitance versus current densities of 3D VNCN-800-SC. (**d**) Ragone plots of 3D VNCN-800-SC.

A symmetrical capacitor based on a 3D VNCN-800 electrode was assembled to evaluate the practical application (marked as 3D VNCN-800-SC). Figure 7a,b show the CV and GCD curves of 3D VNCN-800-SC in the potential window of 0–1.2 V. A pair of reduction/oxidation peaks from the CV curve at about 1.0/1.1 V mainly depends on the rapid redox reaction at V and N active sites, demonstrating the generation of pseudocapacitance supplementing EDLC. Calculated from the GCD curves, the specific capacitance of 3D VNCN-800-SC (Figure 7c) is 78.0 F·g^−1^ (1 A·g^−1^), 72.0 F·g^−1^ (2 A·g^−1^), 66.0 F·g^−1^ (4 A·g^−1^), 63.7 F·g^−1^ (5 A·g^−1^), 58.7 F·g^−1^ (8 A·g^−1^), and 56.7 F·g^−1^ (10 A·g^−1^), respectively. The Ragone plot of 3D VNCN-800-SC (Figure 7d) was plotted based on the GCD curves. A maximum energy density of 15.6 Wh·Kg^−1^ was obtained at a power density of 600 W·Kg^−1^. When the current density increased to 10 A·g^−1^, 3D VNCN-800-SC retains an energy density of 11.3 Wh·Kg^−1^ at a power density of 6003.5 W·Kg^−1^.

## 4. Conclusions

To sum up, we present an effective approach to fabricating 3D VNCN composites. The 3D skeleton comes from polymerized CS-DCDA hydrogel with the assistance of glutaraldehyde. After pyrolysis at high temperatures, the 3D structure is well maintained. Moreover, abundant N dopants and VO_x_ nanoparticles provide additional pseudocapacitance. Hence, 3D VNCN-800 shows a maximum specific capacitance of 408.1 F·g^−1^ at a current density of 1 A·g^−1^ and admirable cycling stability with 96.8% capacitance retention after 5000 cycles. Moreover, 3D VNCN-800-SC delivers a maximum energy density of 15.6 Wh·Kg^−1^ at a power density of 600 W·Kg^−1^. It is hoped that our works could provide guidance for the fabrication of 3D porous carbon materials with excellent electrochemical performance.

## Figures and Tables

**Table 1:** C, N, O, and V element content of 3D VNCN and VNC.

Sample	C (at%)	N (at%)	O (at%)	V (at%)
3D VNCN-700	76.23	9.05	10.72	4.00
3D VNCN-800	80.37	6.95	9.03	3.65
3D VNCN-900	73.97	4.61	13.77	7.65
VNC	77.19	2.96	14.15	5.70

**Table 2:** Electrochemical performances of the as-prepared electrode materials in a three-electrode system.

Materials	C_s_ (1 A·g^−1^, F·g^−1^)	C_s_ (10 Ag^−1^, F·g^−1^)	Retention (1–10 A·g^−1^)
VNC	142.4	78.3	55.0%
3D NCN	172.3	125	72.5%
3D VNCN-700	197.5	115.8	58.6%
3D VNCN-800	408.1	282.5	69.2%
3D VNCN-900	318.6	210.8	66.2%

## Data Availability

The data presented in this study are available on request from the corresponding author.

## Supplementary Materials

The following supporting information can be downloaded at https://www.mdpi.com/article/10.3390/polym15173565/s1, Table S1: Summary of the recently reported performance metrics for 3D porous carbon materials, 3D N-doped porous carbon materials, and 3D porous carbon materials combined with pseudocapacitive materials; Table S2: Summary of the recently reported 3D porous carbon-based materials and their electrochemical performance in three-electrode configurations. Refs. [43,44,45,46,47,48,49,50,51,52] are cited in the supplementary materials.
