# Supplementary material for: 3D Porous VOx/N-Doped Carbon Nanosheet Hybrids Derived from Cross-Linked Dicyandiamide–Chitosan Hydrogels for Superior Supercapacitor Electrode Materials
Source: Polymers (Basel). 2023 Aug 28;15(17):3565. doi: 10.3390/polym15173565 (PMC10490277; doi:10.3390/polym15173565)
Supplement: Supplementary file 1 [file polymers-15-03565-s001.zip › polymers-2555708-supplementary.pdf]

Supporting Information

3D Porous VO_x/N-Doped Carbon Nanosheet Hybrids Derived from Cross-Linked Dicyandiamide–Chitosan Hydrogels for Superior Supercapacitor Electrode Materials

Jinghua Liu [1], Xiong He [1,*], Jiayang Cai [2], Jie Zhou [1], Baosheng Liu [1], Shaohui Zhang [1], Zijun Sun [1], Pingping Su [2], Dezhi Qu [2,*] and Yudong Li [3]

[1] Liuzhou Key Laboratory of New Energy Vehicle Power Lithium Battery, Guangxi Engineering Research Center for Characteristic Metallic Powder Materials, School of Electronic Engineering, Guangxi University of Science and Technology, Liuzhou 545000, China; liujinghua@gxust.edu.cn (J.L.); 17586600924@163.com (J.Z.); liubaosheng@gxust.edu.cn (B.L.); zhangshaohui@gxust.edu.cn (S.Z.); sunzijun@gxust.edu.cn (Z.S.)

[2] Guangxi Key Laboratory of Green Processing of Sugar Resources, College of Biological and Chemical Engineering, Guangxi University of Science and Technology, Liuzhou 545006, China; cjy193677464@163.com (J.C.); m19807720904@163.com (P.S.)

[3] Key Laboratory of Bio-Based Material Science & Technology, Northeast Forestry University, Harbin 150090, China; lydlnn0000@163.com

[*] Correspondence: hexiong@gxust.edu.cn (X.H.); qudezhi199166@gxust.edu.cn (D.Q.)

Table S1 Summary of the recently reported performance metrics for 3D porous carbon materials, 3D N-doped porous carbon materials, and 3D porous carbon materials combined with pseudocapacitive materials.

Materials	SSA (m^2 g^{-1})	N content	Electrolyte	C_s (F g^{-1})	Cycling performance
3D porous carbon					
RHPC [1]	1873	-	6M KOH	263.0 F g^{-1} (0.5 A g^{-1})	95.0% (20000 C, 1 A g^{-1})
PCFs [2]	3301	-	6M KOH	348.0 F g^{-1} (0.2 A g^{-1})	95.0% (10000 C, 20 A g^{-1})
RHAC [3]	3145	-	6M KOH	367.0 F g^{-1} (5 mV s^{-1})	100.0% (30000 C, 2.27A g^{-1})
3D N-doped porous carbon					
3D HPCNS [4]	1777	1.34 wt%	6M KOH	380.0 F g^{-1} (1 A g^{-1})	-
3D NHNC [5]	2090	8.12 wt%	6M KOH	473.5 F g^{-1} (1 A g^{-1})	95.7% (10000 C, 10 A g^{-1})
3D HYPC [6]	1952	1.6 at%	6M KOH	556.0 F g^{-1} (0.5A g^{-1})	94.7% (10000 C, 10 A g^{-1})
3D porous carbon combined with pseudocapacitive material					
3D VN/C [7]	523.5	3.6 at%	6M KOH	392.0 F g^{-1} (1 A g^{-1})	83.5% (5000 C, 2 A g^{-1})
V_2O_5/n-MPC [8]	8.77	2.81 wt%	1 M K_2SO_4	487.0 F g^{-1} (0.5 A g^{-1})	84.0% (2000 C, 10A g^{-1})
3D N-CNFs/V_2O_5 [9]	-	8.57 at%	1 M Na_2SO_4	595.1 F g^{-1} (0.5 A g^{-1})	100.0% (12000 C, 0.5A g^{-1})

Note: SSA is specific surface area; RHPC is rice husk-based hierarchical porous carbon; PCFs are popcorn-derived porous carbon flakes; RHAC is rice husk activated carbon; HPCNS is hierarchical porous carbon nanocages; NHNC is nitrogen-doped hierarchical nanostructure carbon; HYPC is hierarchical porous carbon from yam biowastes; VN/C is vanadium nitride/carbon; V_2O_5/n-MPC is vanadium pentoxide/nitrogen containing mesoporous carbon spheres; N-CNF is N doped carbon nanofiber.

Table S2 Summary of the recently reported 3D porous carbon-based materials and their electrochemical performance in three-electrode configurations.

Materials	Electrolyte	C_s ($F\ g^{-1}$)	Cycling performance
N-doped carbon [10]	6M KOH	244.0 F g^{-1} (1 A g^{-1})	93.0% (5000 C, 5 A g^{-1})
3D N-doped C/CF[11]	6 M KOH	250.6 F g^{-1} (0.5 A g^{-1})	98.4% (10000 C, 2 A g^{-1})
3D VO_x/rGO [12]	1 M Na_2SO_4	252.0 F g^{-1} (1 A g^{-1})	92.0% (10000 C, 100 A g^{-1})
3D V_2O_5/MCHS[13]	1 M Na_2SO_4	313.0 F g^{-1} (0.25 A g^{-1})	81.0% (4000 C, 5 A g^{-1})
3D porous carbon [14]	6 M KOH	320.7 F g^{-1} (1 A g^{-1})	100.0% (10000 C, 5 A g^{-1})
N,O co-doped 3DPC [15]	6 M KOH	352.0 F g^{-1} (0.5 A g^{-1})	99.5% (10000 C, 50 A g^{-1})
3D IHPNCs [16]	1 M H_2SO_4	389.0 F g^{-1} (1 A g^{-1})	90.0% (10000 C, 100 mVs^{-1})
3D VN/C membrane [7]	6 M KOH	392.0 F g^{-1} (1 A g^{-1})	83.5% (5000 C, 2 A g^{-1})
3D PCN@V_2O_5 [17]	1 M Na_2SO_4	457.0 F g^{-1} (0.5 A g^{-1})	88.0% (500 C, 0.5 A g^{-1})
3D NHNC [5]	6 M KOH	473.5 F g^{-1} (1 A g^{-1})	95.7% (10000 C, 10 A g^{-1})
**3D NVCN (this work)**	**6 M KOH**	**408.1 F g^{-1} (1 A g^{-1})**	**96.8% (5000 C, 10 A g^{-1})**

Note: RGO is graphene; MCHS is mesoporous carbon hollow spheres; 3DPC is 3D porous carbon; IHPNCs are interconnected hierarchical porous N-doped carbon nanotubes; PCN is porous g-C_3N_4; NHNC is nitrogen-doped nanostructure carbon.

References

1. Chen, Z.; Wang, X.; Xue, B.; Li, W.; Ding, Z.; Yang, X.; Qiu, J.; Wang, Z., Rice husk-based hierarchical porous carbon for high performance supercapacitors: The structure-performance relationship. *Carbon* **2020,** 161, 432-444.
2. Hou, J.; Jiang, K.; Wei, R.; Tahir, M.; Wu, X.; Shen, M.; Wang, X.; Cao, C., Popcorn-derived porous carbon flakes with an ultrahigh specific surface area for superior performance supercapacitors. *ACS Applied Materials & Interfaces* **2017,** 9, (36), 30626-30634.
3. Gao, Y.; Li, L.; Jin, Y.; Wang, Y.; Yuan, C.; Wei, Y.; Chen, G.; Ge, J.; Lu, H., Porous carbon made from rice husk as electrode material for electrochemical double layer capacitor. *Applied Energy* **2015,** 153, 41-47.
4. Shao, J.; Song, M.; Wu, G.; Zhou, Y.; Wan, J.; Ren, X.; Ma, F., 3D carbon nanocage networks with multiscale pores for high-rate supercapacitors by flower-like template and in-situ coating. *Energy Storage Materials* **2018,** 13, 57-65.
5. Shang, Z.; An, X.; Zhang, H.; Shen, M.; Baker, F.; Liu, Y.; Liu, L.; Yang, J.; Cao, H.; Xu, Q.; Liu, H.; Ni, Y., Houttuynia-derived nitrogen-doped hierarchically porous carbon for high-performance supercapacitor. *Carbon* **2020,** 161, 62-70.
6. Li, Z.; Liu, Q.; Sun, L.; Li, N.; Wang, X.; Wang, Q.; Zhang, D.; Wang, B., Hydrothermal synthesis of 3D hierarchical ordered porous carbon from yam biowastes for enhanced supercapacitor performance. *Chemical Engineering Science* **2022,** 252, 117514.
7. Wu, Y.; Yang, Y.; Zhao, X.; Tan, Y.; Liu, Y.; Wang, Z.; Ran, F., A novel hierarchical porous 3D structured vanadium nitride/carbon membranes for high-performance supercapacitor negative electrodes. *Nano-Micro Letters* **2018,** 10, 1-11.
8. Saravanakumar, B.; Purushothaman, K. K.; Muralidharan, G., V_2O_5/nitrogen enriched mesoporous carbon spheres nanocomposite as supercapacitor electrode. *Microporous and Mesoporous Materials* **2018,** 258, 83-94.
9. Sun, W.; Gao, G.; Zhang, K.; Liu, Y.; Wu, G., Self-assembled 3D N-CNFs/V_2O_5 aerogels with core/shell nanostructures through vacancies control and seeds growth as an outstanding supercapacitor electrode material. *Carbon* **2018,** 132, 667-677.
10. Ping, Y.; Yang, S.; Han, J.; Li, X.; Zhang, H.; Xiong, B.; Fang, P.; He, C., N-self-doped graphitic carbon aerogels derived from metal–organic frameworks as supercapacitor electrode materials with high-performance. *Electrochimica Acta* **2021,** 380, 138237.
11. Shi, C.; Li, S.; Pan, Y.; Guo, L.; Wang, Y., Self-standing porous N doped carbon/carbon foam for high-performance supercarpacitor. *Diamond and Related Materials* **2020,** 110, 108138.
12. Zhang, L. F.; Tang, J.; Liu, S. Y.; Peng, O. W.; Shi, R.; Chandrashekar, B. N.; Li, Y.; Li, X.; Li, X. N.; Xu, B. M.; Cheng, C., A laser irradiation synthesis of strongly-coupled VO_x-reduced graphene oxide composites as enhanced performance supercapacitor electrodes. *Materials Today Energy* **2017,** 5, 222-229.
13. Zhang, G.; Ren, L.; Hu, D.; Zhang, S.; Gu, H., Fabrication of mesoporous carbon hollow spheres intercalated three-dimensional network structure V_2O_5 nanosheets with enhanced electrochemical performance. *Journal of Alloys and Compounds* **2019,** 781, 407-414.
14. Geng, D.; Zhang, S.; Jiang, Y.; Jiang, Z.; Shi, M.; Chang, J.; Liang, S.; Zhang, M.; Feng, J.; Wei, T., 3D interconnected porous carbon derived from spontaneous merging of the nano-

sized ZIF-8 polyhedrons for high-mass-loading supercapacitor electrodes. *Journal of Materials Chemistry A* **2022,** 10, (4), 2027-2034.

15. Liu, H.; Huang, X.; Zhou, M.; Gu, J.; Xu, M.; Jiang, L.; Zheng, M.; Li, S.; Miao, Z., Efficient conversion of biomass waste to N/O co-doped hierarchical porous carbon for high performance supercapacitors. *Journal of Analytical and Applied Pyrolysis* **2023,** 169, 105844.
16. Zuo, S.; Chen, J.; Liu, W.; Li, X.; Kong, Y.; Yao, C.; Fu, Y., Preparation of 3D interconnected hierarchical porous N-doped carbon nanotubes. *Carbon* **2018,** 129, 199-206.
17. Zhou, Y.; Sun, L.; Wu, D.; Li, X.; Li, J.; Huo, P.; Wang, H.; Yan, Y., Preparation of 3D porous g-C_3N_4@V_2O_5 composite electrode via simple calcination and chemical precipitation for supercapacitors. *Journal of Alloys and Compounds* **2020,** 817, 152707.
